# Causal Effects of Time-Dependent Treatments in Older Patients with Non-Small Cell Lung Cancer

**DOI:** 10.1371/journal.pone.0121406

**Published:** 2015-04-07

**Authors:** Igor Akushevich, Konstantin Arbeev, Julia Kravchenko, Mark Berry

**Affiliations:** 1 Center for Population Health and Aging, Duke University, Durham, North Carolina, United States of America; 2 Department of Surgery, Duke University Medical Center, Durham, North Carolina, United States of America; 3 Department of Cardiothoracic Surgery, Stanford University, Stanford, California, United States of America; IPO, Portuguese Institute of Oncology of Porto, PORTUGAL

## Abstract

**Background:**

Treatment selection for elderly patients with lung cancer must balance the benefits of curative/life-prolonging therapy and the risks of increased mortality due to comorbidities. Lung cancer trials generally exclude patients with comorbidities and current treatment guidelines do not specifically consider comorbidities, so treatment decisions are usually made on subjective individual-case basis.

**Methods:**

Impacts of surgery, radiation, and chemotherapy mono-treatment as well as combined chemo/radiation on one-year overall survival (compared to no-treatment) are studied for stage-specific lung cancer in 65+ y.o. patients. Methods of causal inference such as propensity score with inverse probability weighting (IPW) for time-independent and marginal structural model (MSM) for time-dependent treatments are applied to SEER-Medicare data considering the presence of comorbid diseases.

**Results:**

122,822 patients with stage I (26.8%), II (4.5%), IIIa (11.5%), IIIb (19.9%), and IV (37.4%) lung cancer were selected. Younger age, smaller tumor size, and fewer baseline comorbidities predict better survival. Impacts of radio- and chemotherapy increased and impact of surgery decreased with more advanced cancer stages. The effects of all therapies became weaker after adjustment for selection bias, however, the changes in the effects were minor likely due to the weak selection bias or incompleteness of the list of predictors that impacted treatment choice. MSM provides more realistic estimates of treatment effects than the IPW approach for time-independent treatment.

**Conclusions:**

Causal inference methods provide substantive results on treatment choice and survival of older lung cancer patients with realistic expectations of potential benefits of specific treatments. Applications of these models to specific subsets of patients can aid in the development of practical guidelines that help optimize lung cancer treatment based on individual patient characteristics.

## Introduction

Lung cancer is the leading cause of cancer mortality in the United States and primarily occurs in older adults, with an approximate average age of 69 years at diagnosis. Clinicians must make lung cancer therapy decisions by weighing the *pro* benefits of curative and life-prolonging therapy against *contra* factors such as increased mortality risk due to comorbid conditions. Unfortunately, estimating both the risks and benefits of treatment for older patients is difficult. Trials evaluating lung cancer treatments often exclude elderly patients to avoid an obscuration of the cancer treatment effects by patients’ comorbid conditions [1–4]. The presence and severity of comorbid conditions in elderly patients are generally known to increase the risk of treatment toxicity and decrease treatment tolerance; however, the data that more specifically guides therapies are severely lacking [5]. In the end, existing guidelines do not provide detailed information that can help to make these difficult decisions and treatment is essentially guided by subjective clinical judgment on an individual case basis [6].

Recent advances in collection of powerful datasets and in development of statistical methods such as causal inference give researchers new opportunities to precisely compare the effect of different treatment modes for minimally heterogeneous groups of patients. The analysis of the linked Surveillance, Epidemiology, and End Results (SEER)-Medicare database by methods allowing for addressing the selection bias (the most important challenge in analysis of observational data) could provide new and comprehensive information about treatment modes that can be time-dependent. Using these analyses for relatively homogenous subsets of patients based on individual characteristics such as cancer stage, treatment, and comorbid conditions can potentially greatly aid in the development of treatment guidelines in circumstances where strong quantitative evidence is currently lacking. However, the methods of causal inference have been never applied to SEER-Medicare data and their ability to provide the causal estimates (as well as the properties of these estimates) is not known. The first and inevitable step in addressing this gap in knowledge is to check how standard approaches of causal inference for time-independent and time-dependent treatments which were successfully applied in other areas of medical research could work in cancer research. This step is the main focus of this study.

Propensity-score-based approaches (e.g., inverse probability weighting (IPW)) and marginal structural models (MSMs) are currently the most successful statistical technologies capable of addressing selection bias for time-independent and time-dependent treatments, respectively [7,8]. MSM uses the IPW approach to evaluate individual (stabilized) weights and then evaluates the effects of time-dependent treatments within a weighted repeated measure approach. MSM has been used in several circumstances [9–11] but its use for cancer treatment has not been reported. The objective of this analysis is to apply IPW and MSM to SEER-Medicare data to study the causal effects of treatment (surgery, radiation, or chemotherapy, as well as no treatment) on survival of patients with lung cancer given individual patient’s tumor characteristics, comorbidities, and demographic and socioeconomic factors. Specific attention is paid to dynamic interrelations of treatment and comorbidities, given that comorbidity impacts both treatment choice and treatment effectiveness while cancer therapy can aggravate co-existing conditions. Methodologically, we investigate how applying the methods of causal inference to large scale observational data such as SEER-Medicare can help clarify the effects of different treatment modalities on lung cancer survival.

## Data and Methods

The expanded SEER registry covers approximately 26% of the U.S. population. The Medicare records for several millions of individuals are available in SEER-Medicare including 413,776 individuals with lung cancer. For the majority of patients, continuous records of Medicare services use are available from 1991 (or from the time the person has passed the age of 65 after 1990) until the patient's death. A small fraction of individuals (e.g., new patients diagnosed with cancer in 2003–2007) has Medicare records from 1998. The Medicare records are available for each institutional (MedPAR, outpatient, hospice, or home health agency (HHA)) and non-institutional (Carrier-Physician-Supplier and Durable Medical Equipment Providers) claim type.

Treatment patterns (i.e., the prevalence of each treatment type including chemotherapy, radiation therapy, and surgery at each day of individual follow-up) are constructed using ICD-9, CPT/HCPCS, and revenue centers procedure codes available in different Medicare sources. The approach to reconstruct the date at onset is similar to that used in Berry et al. [12]. Information from i) demographic characteristics (age, sex, and race), ii) tumor-related characteristics (histology, stage, and TNM status), iii) area-based social-economic statuses (SES), and iv) prevalence of other diseases reflected in the comorbidity index, is used to create baseline and time-dependent (for comorbidity only) predictors of treatment mode and survival. Socio-economic factors are represented by census tract based information on patient’s residence; this information is obtained from the 1990 or 2000 U.S. census bureau surveys, depending on the patient’s year of cancer diagnosis, respectively. The following SES variables are considered: percentage of blacks, percentage of persons aged 25+ years old who has at least four years of college education, and percentage of the residents living below the poverty level. Dynamically changing comorbidity status is represented by the comorbidity index calculated as *C*(*t*) = Σ_*d*_ *w*_*d*_ *I*_*d*_(*t*), where *I*_*d*_(*t*) are the indicators of diseases at time *t*, and *w*_*d*_ are disease weights estimated using the Cox regression model applied to the entire cohort of lung cancer patients, controlling by patients’ age, race, sex and stage at diagnosis. The details of the calculation and the list of 85 conditions contributing to the index are discussed in Kravchenko et al., [13]. In the present paper, the comorbidity index is categorized into five groups based on percentiles of its distribution for all patients selected for the analysis. Patients in Group 0 had the least amount of co-morbidities while patients in Group 4 had the highest amount of co-morbidities.

The following inclusion criteria were used: i) lung cancer diagnosis was made during the period of time from 1992 to 2007; ii) the age at diagnosis was 65+ years; iii) tumor histological type was non-small cell carcinoma; iv) patients had health insurance coverage by Part A and B Medicare and no HMO insurance in each month of the period from 12 month before and 6 month after the diagnosis; v) tumor stage at the time of diagnosis as defined using the Modified AJCC Stage 3^rd^ (1992–2003) and 6^th^ editions was either stages I, II, IIIA, IIIB, and IV and not classified as “unknown”; vi) the date of lung cancer onset as identified from the analysis of Medicare trajectories [14] fell into the period not earlier than two and not later than three months compared with the SEER recorded date of cancer diagnosis; vii) information about the three SES variables described above (SES black, SES college, and SES poverty) is not missing; viii) the death event did not occur earlier than 15 days from lung cancer diagnosis; and ix) tumor stage T was not stage T0.

The methods of causal inference are used to evaluate the effects of treatment on survival of lung cancer patients in stage-specific strata. For time-independent treatments (represented by a non-ordered list of treatments applied to a patient), we used the propensity score approach with inverse probability weighting methodologically following the computational scheme used in [15]. The components of this approach are i) the estimation of treatment model and evaluation of individual weights, ii) checking the quality of created pseudorandomization by analysis of the tables that compare variables among treatment groups for original and weighted (i.e., pseudorandomized) patient cohorts, and iii) evaluation of the treatment effect for the weighted cohorts and its comparison with the estimate obtained without using the weights. The methods were then generalized for use with time-dependent treatments. Such approaches are known as the marginal structural models [7,8]. In this approach, IPWs were first calculated for each time point using both baseline and time-dependent predictors. The estimates of treatment effects were then obtained with a weighted repeated measures approach when both parameters responsible for treatment effect and controlling factors as well as the parameters of correlation matrix capturing the effect of different time points were estimated.

Ethics statement. The data used in this study have no individual identifiable information. No written informed consent given by participants and no specific procedures for the de-identification of the records were required. All data analyses were designed and performed in accordance with the ethical standards of the responsible committee on human experimentation and with the Helsinki Declaration (of 1975, revised in 1983) and have been approved by the Duke University Health System Institutional Review Board (Pro00030031).

## Analysis and Results

The baseline characteristics of selected patients are given in Table 1. In total, we selected 122,822 lung cancer patients of stages I (26.8%), II (4.5%), IIIa (11.5%), IIIb (19.9%), and IV (37.4%). As seen in Table 1, the age group of 70–74 years has the highest number of diagnoses and the distribution of ages at diagnoses is similar for all stages. Overall males are diagnosed more often than females. Females are more often diagnosed at earlier stages. In contrast, non-white patients are more often diagnosed at higher stages. Both adenocarcinoma (AC) and squamous cell carcinoma (SCC) of the lung are diagnosed more often at earlier stages compared to other lung cancer histotypes. The shapes of the distributions of T and N statuses are expected from clinical perspective (M-status is not shown because it is M1 for stage IV and M0 for other stages). Patients diagnosed at more advanced cancer stages had more comorbidities. As would be expected based on current treatment guidelines and practice, the prevalence of surgery dramatically drops among patients with late-stage lung cancer. In contrast, treatments involving chemo- and radiation therapy (as well as “no treatment” option) are used more often in therapies of advanced cancers. Patients with higher SES (whose living area is characterized by more educated, lower-poverty-level, and lower-fraction-of-blacks population) are diagnosed at earlier stages, though the effect is minor.

**Table 1:** Demographic, tumor, socio-economic characteristic and treatment modes for lung cancer patients with stages I, II, IIIA, IIIB, and IV.

Variable	I	II	IIIA	IIIB	IV	Total
Stage	32895 (26.8)	5480 (4.5)	14095 (11.5)	24383 (19.9)	45969 (37.4)	122822 (100)
Age (years)						
65–69	6571 (20.0)	1315 (24.0)	2956 (21.0)	4519 (18.5)	9775 (21.3)	25136 (20.5)
70–74	9509 (28.9)	1710 (31.2)	4081 (29.0)	6486 (26.6)	12835 (27.9)	34621 (28.2)
75–79	8879 (27.0)	1437 (26.2)	3713 (26.3)	6122 (25.1)	11858 (25.8)	32009 (26.1)
80–84	5433 (16.5)	758 (13.8)	2300 (16.3)	4395 (18.0)	7549 (16.4)	20435 (16.6)
85+	2503 (7.6)	260 (4.7)	1045 (7.4)	2861 (11.7)	3952 (8.6)	10621 (8.6)
Race						
White	30767 (93.5)	5160 (94.2)	12977 (92.1)	22150 (90.8)	42049 (91.5)	113103 (92.1)
Non-White	2128 (6.5)	320 (5.8)	1118 (7.9)	2233 (9.2)	3920 (8.5)	9719 (7.9)
Sex						
Male	16690 (50.7)	3072 (56.1)	8010 (56.8)	13616 (55.8)	25266 (55.0)	66654 (54.3)
Female	16205 (49.3)	2408 (43.9)	6085 (43.2)	10767 (44.2)	20703 (45.0)	56168 (45.7)
Histology						
AC	13948 (42.4)	2225 (40.6)	4262 (30.2)	8780 (36.0)	17343 (37.7)	46558 (37.9)
SCC	10422 (31.7)	1926 (35.1)	4942 (35.1)	6663 (27.3)	8149 (17.7)	32102 (26.1)
Other	8525 (25.9)	1329 (24.3)	4891 (34.7)	8940 (36.7)	20477 (44.5)	44162 (36.0)
T-status						
T1	14558 (44.3)	1369 (25.0)	2320 (16.5)	333 (1.4)	7044 (15.3)	25624 (20.9)
T2	14981 (45.5)	3149 (57.5)	5473 (38.8)	659 (2.7)	14425 (31.4)	38687 (31.5)
T3	14 (0.0)^§^	633 (11.6)	3495 (24.8)	586 (2.4)	909 (2.0)	5637 (4.6)
T4				14777 (60.6)	7423 (16.1)	22200 (18.1)
TX	3342 (10.2)	329 (6.0)	2807 (19.9)	8028 (32.9)	16168 (35.2)	30674 (25.0)
N-status						
N0	29347 (89.2)	628 (11.5)	1560 (11.1)^§^	5959 (24.4)	8404 (18.3)	45898 (37.4)
N1		4405 (80.4)	519 (3.7)	1233 (5.1)	2124 (4.6)	8281 (6.7)
N2			11111 (78.8)	8352 (34.3)	15703 (34.2)	35166 (28.6)
N3				3049 (12.5)	3486 (7.6)	6535 (5.3)
NX	3548 (10.8)	447 (8.2)	905 (6.4)	5790 (23.7)	16252 (35.4)	26942 (21.9)
Comorbidity*						
0	11987 (36.4)	1851 (33.8)	3712 (26.3)	4265 (17.5)	6457 (14.0)	28272 (23.0)
1	8543 (26.0)	1476 (26.9)	3571 (25.3)	4882 (20.0)	8058 (17.5)	26530 (21.6)
2	6192 (18.8)	1105 (20.2)	3123 (22.2)	5203 (21.3)	9501 (20.7)	25124 (20.5)
3	4039 (12.3)	699 (12.8)	2325 (16.5)	5265 (21.6)	10896 (23.7)	23224 (18.9)
4	2134 (6.5)	349 (6.4)	1364 (9.7)	4768 (19.6)	11057 (24.1)	19672 (16.0)
Treatment**						
No	5177 (15.7)	485 (8.9)	2531 (18.0)	7939 (32.6)	15475 (33.7)	31607 (25.7)
Che	804 (2.4)	137 (2.5)	947 (6.7)	3349 (13.7)	8643 (18.8)	13880 (11.3)
Rad	3979 (12.1)	448 (8.2)	2494 (17.7)	3539 (14.5)	6461 (14.1)	16921 (13.8)
Che+Rad	2546 (7.7)	669 (12.2)	4455 (31.6)	7010 (28.7)	13737 (29.9)	28417 (23.1)
Sur	17415 (52.9)	1839 (33.6)	1292 (9.2)	1180 (4.8)	633 (1.4)	22359 (18.2)
Sur+Che	1189 (3.6)	587 (10.7)	483 (3.4)	389 (1.6)	334 (0.7)	2982 (2.4)
Sur+Rad	1228 (3.7)	784 (14.3)	878 (6.2)	405 (1.7)	251 (0.5)	3546 (2.9)
Sur+Che+Rad	557 (1.7)	531 (9.7)	1015 (7.2)	572 (2.3)	435 (0.9)	3110 (2.5)
SES (Mean,STD)						
SES black	9.2 (19.8)	8.5 (18.6)	9.9 (20.9)	10.6 (21.7)	10.3 (21.4)	9.9 (20.9)
SES college	24.9 (16.6)	25.0 (16.3)	24.0 (16.0)	23.5 (16.0)	24.1 (16.2)	24.2 (16.2)
SES poverty	10.8 (9.7)	10.5 (9.4)	11.2 (9.8)	11.8 (10.2)	11.4 (10.1)	11.3 (9.9)

*—quintiles of distribution of entire patient groups, i.e., for all stages (0—lowest, 4—highest)

**—chemotherapy (Che), radiation therapy (Rad), and surgery (Sur)

^§^-there are some minor inconsistencies in the recorded T and N statuses and respective AJCC stage (e.g., T3N0 should be stage II; T1-3N0 would be either stage I or stage II, not IIIA).

Values in brackets are percents of all patients (for Stage, i.e., first line of the Table) or stage-specific cohorts of patients.

### Time-independent treatment

**Fig 1:**
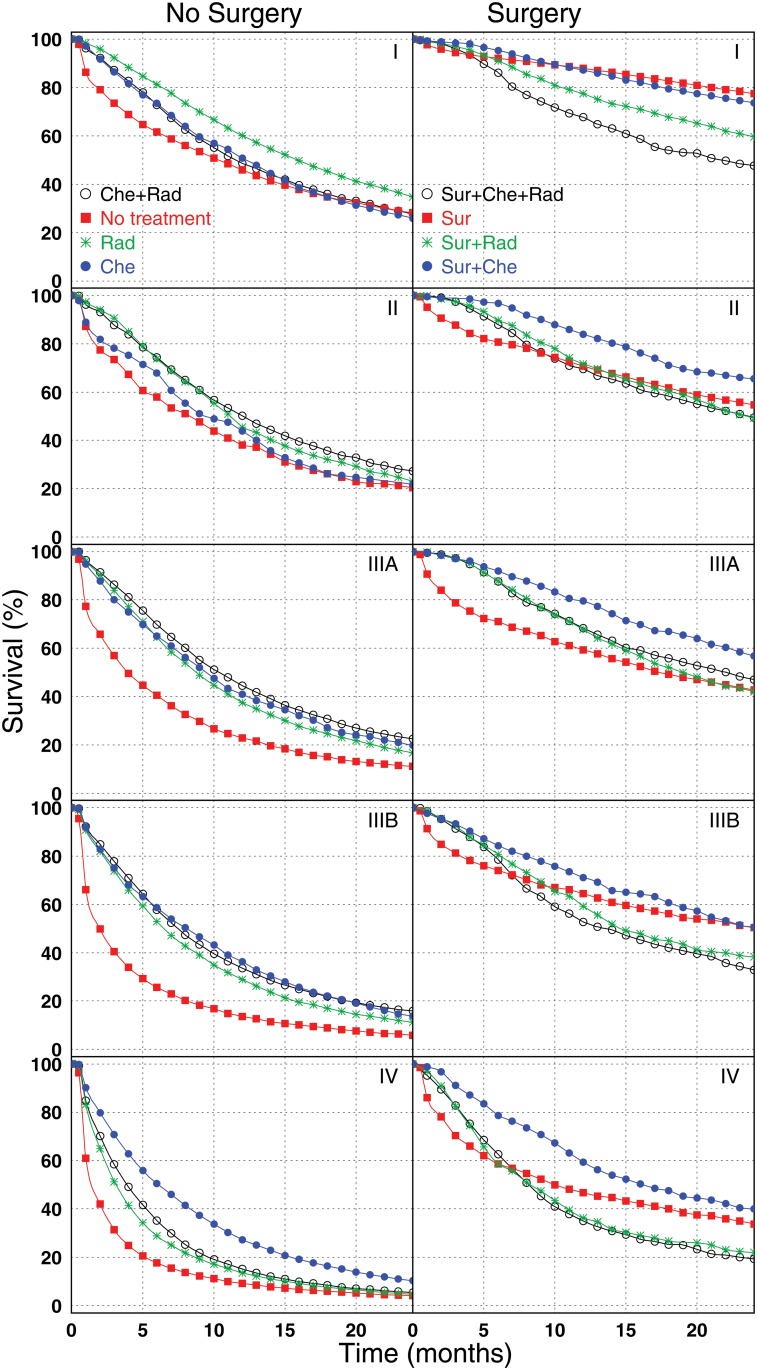
Survival curves for treatment-specific and stage-specific patient groups. Rows correspond to stages I, II, IIIA, IIIB, and IV.

Treatment modes are defined using information from procedure codes in the time period from the date at diagnosis to 200 days after the diagnosis. Occurrence of any code associated with surgery, chemotherapy, or radiotherapy in any Medicare file indicates the respective treatment for a patient. Two-year stage- and treatment-specific survival functions are presented in Fig 1. Left columns show the effects for treatment modes not involving surgery, including no therapy at all, while the right columns show the effects of treatments of surgery with or without other therapies. Several conclusions can be made from qualitative review of the treatment-specific survival functions. Treatments involving surgery are beneficial for each stage. At least in part, this could be due to a selection bias where surgery was preferentially selected for patients who were healthier. Patients who underwent surgery may differ by specific tumor characteristics. For example, while surgery is very unlikely used for patients with widespread metastases, it is sometimes used for stage IV patients who only have one site of metastases. Also, for stage IIIA, surgery is more likely used for patients whose N2 disease is due to a limited amount of microscopic findings as compared to the patients who have extensive metastases in lymph nodes. For stage I patients, adding other therapies to surgery did not appear to provide additional short-term survival benefit. For higher stage patients, combining surgery with other therapies appeared to improve survival. In addition, early survival for patients who had surgery for stages II-IV appears worst for patients who only had surgery—this finding could be due to patients who were primarily treated with surgery and had complications or mortality from surgery that limited their ability to be given other therapies.

Selection bias can be addressed by using the propensity score based analysis with IPW. The treatment model (generalized logit model) predicting the probability to have one of eight treatment modes (i.e., any combination of surgery, chemo-, and radiotherapy vs. no treatment) is estimated controlling by sex, race, age group, T-status, three SES variables (categorized into the three groups according to percentiles), comorbidity index, and histology. The model predicts probabilities to have any treatment for each patient. Individual weights are then calculated as reciprocal of probability to have actually observed treatment, resulting in a weighted population that is pseudorandomized with respect to health-related characteristics for subcohorts by each administered treatment mode. Table 2 and S1 and S2 Tables show that frequency distributions evaluated for weighted population are similar for all treatment-specific subcohorts: p-values of the formal tests checking the distributions among treatment groups are collected in Table 2 and the complete information (including actual numbers of patients in treatment groups and percentage calculated without and with the weights) is presented in S1 and S2 Tables. The results show that although almost all variables are distributed differently in the patient groups, this heterogeneity disappears for pseudorandomized cohorts for which respective percentages and p-values are calculated using the IP weights.

**Table 2:** The p-values of the *χ* ^2^-tests for treatment-group comparison of stage-specific cohorts of lung cancer patients calculated for original and pseudorandomized (i.e., weighted) populations.

Stage	I	I	II	II	IIIA	IIIA	IIIB	IIIB	IV	IV
Weight	No	IP	No	IP	No	IP	No	IP	No	IP
Sex	<.0001	0.9877	0.3944	0.6365	0.0073	0.9926	<.0001	0.3624	<.0001	0.1569
Race	<.0001	0.8535	<.0001	0.9867	<.0001	0.5864	<.0001	0.0737	<.0001	0.8942
Age	<.0001	0.9945	<.0001	0.8566	<.0001	0.9771	<.0001	0.9985	<.0001	0.4444
T-status	<.0001	0.9945	<.0001	0.9932	<.0001	0.9783	<.0001	0.4964	<.0001	0.1920
SES(black)	<.0001	0.9898	<.0001	0.9999	0.0008	0.9523	0.0050	0.9310	<.0001	0.1989
SES(college)	<.0001	0.6077	<.0001	0.9938	<.0001	1.0000	<.0001	0.9799	<.0001	0.4817
SES(poverty)	<.0001	0.3820	<.0001	0.8949	<.0001	0.7707	<.0001	0.3551	<.0001	0.9934
Histology	<.0001	0.4553	<.0001	0.9919	<.0001	0.9469	<.0001	0.9473	<.0001	0.0107
Comorbidity	<.0001	0.4361	<.0001	0.9788	<.0001	0.6010	<.0001	0.2881	<.0001	0.0773

The causal effect of the treatment modes is evaluated in the Cox model for the pseudorandomized population. The results of the analysis for one-year survival are presented in Table 3. We right-censored all patients who had not died and had follow-up beyond one year at the one year time point. Both weighted and non-weighted estimates are presented. The main predictor of interest was the eight-category variable representing treatment modes. Three cofactors were used: age group, comorbidity group, and T-status. The evaluated effects of these cofactors are expected: evident increase of their effects for age group, comorbid subgroups or subgroups with higher values of T-status. The effects of treatment modes are also expected and in concordance with the results shown in Fig 1. The hazard ratio (HR) of surgery decreases for higher stages, while the HR of radiation and/or chemotherapy increases for higher stages. The reproduction of results expected from clinical experience is the first observation from the estimates presented in Table 3. The second observation is that the estimates of the treatment effects do not change strongly for original and pseudorandomized patient groups. This observation suggests that the selection bias is not as strong as initially suspected or that the set of observed variables in Table 2 do not adequately cover the actual list of variables predicting the treatment choice. The third observation is that the effects of all treatments (vs. no treatment) became smaller in the pseudorandomized population for all comparisons except the subcohort of stage IV patients treated with both surgery and radiotherapy.

**Table 3:** The causal effect of the lung cancer treatment modes (represented by HRs) evaluated in the Cox model for original and pseudorandomized population for one-year follow-up for treatments involving surgery (Sur), radio- (Rad) and chemotherapy (Che) vs. no treatment.

Stage	I	I	II	II	IIIA	IIIA	IIIB	IIIB	IV	IV
Weight	No	IP	No	IP	No	IP	No	IP	No	IP
Treatment										
Che	0.842	0.924	1.0**	1.13*	0.625	0.648	0.500	0.514	0.495	0.503
Rad	0.667	0.753	0.758	0.836	0.604	0.637	0.615	0.621	0.778	0.783
Che+Rad	0.880	1.0**	0.711	0.784	0.557	0.585	0.581	0.606	0.702	0.718
Sur	0.249	0.285	0.438	0.496	0.420	0.521	0.304	0.423	0.398	0.521
Sur+Che	0.246	0.352	0.229	0.233	0.201	0.211	0.225	0.370	0.247	0.254
Sur+Rad	0.414	0.469	0.399	0.471	0.289	0.352	0.314	0.333	0.436	0.394
Sur+Che+Rad	0.621	0.763	0.459	0.561	0.308	0.341	0.383	0.445	0.452	0.565
Age (years)										
70–74 vs. 65–69	1.163	1.208	1.151	1.144	1.131	1.194	1.098	1.107	1.031	1.112
75–79 vs. 65–69	1.276	1.243	1.303	1.204	1.306	1.373	1.147	1.04*	1.111	1.237
80–84 vs. 65–69	1.437	1.519	1.415	1.648	1.412	1.481	1.248	1.311	1.143	1.195
85+ vs. 65–69	1.534	1.925	1.697	2.067	1.505	1.608	1.219	1.649	1.110	1.574
T-status										
T2 vs. T1	1.695	1.519	1.545	1.389	1.460	1.516	1.248	1.703	1.223	1.325
T3 vs. T1	1.2**	0.4**	1.867	1.816	1.785	1.835	1.649	1.928	1.269	1.471
T4 vs. T1							1.409	1.765	1.211	1.323
TX vs. T1	2.094	2.110	1.454	1.274	1.845	2.096	1.741	2.454	1.295	1.297
Comorbidity										
1 vs. 0	1.350	1.349	1.295	1.336	1.219	1.228	1.306	1.377	1.209	1.217
2 vs. 0	1.769	1.705	1.512	1.677	1.465	1.527	1.553	1.491	1.493	1.534
3 vs. 0	2.322	2.202	1.861	2.042	1.886	1.903	1.908	2.152	1.862	1.792
4 vs. 0	3.486	3.688	3.315	3.402	2.733	2.934	2.660	2.637	2.687	2.944

*—estimate is not significant (0.05<p<0.3)

**—estimate is not significant (0.3≤p)

### Time-dependent treatment

One limitation in using time-independent treatments is that specific treatment can be not assigned to an individual because of his/her death. This can distort the effects of specific treatment on survival. Moreover, another conclusion from Fig 1 is that the Cox proportional hazard model might not work for the entire time period of individual follow-up. Therefore, a longitudinal model for repeated data in which the probabilities of treatment are evaluated and survival during short period of time is considered, could be better applicable. Such approach is known as MSM [7,8], the logistic model for weighted repeated measures model with generalized estimating equations (GEE). In this model, probability of different treatment modes of interest are modeled for preselected time points of individual follow-up (e.g., each two months). Pseudorandomization using baseline and time-dependent variables is created at each time point. The survival probability is then modeled for each time point and observations for the same patients are considered as repeated measurements. The results for two treatment groups (involving and not involving surgery) are presented in Table 4. Table 4 also contains the estimates of HR for time-independent treatments (as in Table 3, but selecting or unselecting patients with surgery). One observation from the results is that beneficial effects of chemo- and radiotherapy are more pronounced for advanced stages of lung cancer. Although odds ratios (OR) calculated without using the IP weights are at the level of 1.0 (or even higher) even for advanced stages, incorporation of IP weights results in significant beneficial effects for stages IIIA,B and IV. Another observation is that there exist situations when treatment can be harmful: both OR for MSM and HR calculated for time-independent treatments could be around or exceed 2.0 when all three treatments are administered for patients with stage I and II––these findings suggest that overtreatment in some situations expose patients to morbidity and mortality secondary to the treatment without providing additional survival benefit. Also, we see that estimates for time-independent treatment show more positive benefits than those obtained within MSM. The difference in these estimates comes from the contributions of person-months without chemo- or radiotherapy: for MSM these person-months are considered as no treatment control, while for time-independent treatment they contribute to the treatment estimated for this individual. For the cases without surgery these person-years without treatment in this month correspond to better survival, therefore, we observe more positive benefits for time-independent treatments. Similar arguments allow us to understand the differences that are observed for the patients treated with surgery. We see a similar picture for advanced stages because “surgery only” is not optimal treatment strategy for advanced stages and we have opposite situation for stage I because “surgery only” is the most optimal treatment for stage I (see Fig 1).

**Table 4:** MSM estimates for two treatment groups (involving and not involving surgery) of lung cancer patients represented by ORs and respective IPW estimates for time-independent treatments represented by HRs.

Stage	I	I	II	II	IIIA	IIIA	IIIB	IIIB	IV	IV
Weight	No	IP	No	IP	No	IP	No	IP	No	IP
Patients without surgery (vs. no treatment)										
Methods: MSM for time-dependent treatment (OR)										
Che+Rad	1.055	0.998	1.174	1.014	1.094	0.920	1.044	0.849	1.237	0.949
Che	1.043	0.989	1.057	1.023	0.969	0.680	0.979	0.667	1.159	0.688
Rad	0.988	0.733	1.045	0.906	1.026	0.799	1.028	0.792	1.096	0.889
Methods: IP for time-independent treatment (HR)										
Che+Rad	0.851	0.870	0.721	0.747	0.557	0.587	0.576	0.603	0.702	0.718
Che	0.813	0.795	1.0**	1.1**	0.625	0.651	0.498	0.513	0.496	0.505
Rad	0.655	0.649	0.758	0.772	0.604	0.633	0.613	0.618	0.778	0.782
Patients with surgery (vs. surgery only)										
Methods: MSM for time-dependent treatment (OR)										
Sur+Che+Rad	1.483	2.741	1.301	1.959	1.193	1.447	1.226	0.905	1.106	0.946
Sur+Che	1.200	1.593	1.119	0.914	1.126	0.880	1.154	0.991	1.204	0.804
Sur+Rad	1.302	1.920	1.092	1.202	1.140	1.030	1.193	1.031	1.118	1.090
Methods: IP for time-independent treatment (HR)										
Sur+Che+Rad	2.602	2.569	1.0**	1.12*	0.725	0.739	1.262	1.16*	1.11*	1.13*
Sur+Che	1.0**	1.134	0.515	0.513	0.479	0.481	0.751	0.788	0.596	0.584
Sur+Rad	1.682	1.715	0.91*	0.9**	0.692	0.702	1.0**	1.0**	1.11*	1.13*

*—estimate is not significant (0.05<p<0.3)

**—estimate is not significant (0.3≤p)

The standard formulation of MSM requires using so-called stabilized weights that are calculated as: Π_*t*_(*w*_*t*_ */w*_*t0*_), where index *t* runs over all time periods (including baseline, i.e., the month of diagnosis), and time-specific weights *w*_*t*_ and *w*_*t0*_ represent reciprocal of probabilities of actually observed treatments conditional on baseline predictors *c*_*b*_ with or without time-dependent predictors *c*_*t*_, i.e., *w*_*t*_ = [Pr(*T* = *T*_*t*_|*c*_*b*_, *c*_*t*_)]^-1^ and *w*_*t0*_ = [Pr(*T* = *T*_*t*_|*c*_*b*_)]^-1^. The calculation of the stabilized weights involves two additional specific approaches compared to the approach for calculating IP weights for time-independent treatments (i.e., simply *w*_*t*_ for a time point): i) the weights in a certain time point are calculated as a ratio *w*_*t*_ */w*_*t0*_, i.e., an additional factor is used in the denominator and ii) the weights are calculated as products of the weights obtained during measurements at previous time points. We do not use both types of adjustments for the weights calculation in our approach and use the usual formula for the weight *w*_*t*_ = [Pr(*T* = *T*_*t*_|*c*_*b*_, *c*_*t*_)]^-1^with tumor characteristics, current comorbidity index, previous treatment, SES factor and demography (sex and age) as predictors of specific treatments. The choice was based on the comparison of the results obtained using this approach and approaches based on the stabilized weights, and non-stabilized weights with multiplications over previous time points. Only our chosen approach provided reasonable pseudorandomization in all considered time points, which is illustrated in S3 Table. The pseudorandomization in both of the two alternative approaches described is not sufficient, resulting in occurrence of a bias in the survival model parameter estimates. For example, the effect of chemotherapy in Table 4 (weights are *w*_*t*_) is OR = 0.989 for stage I, while the approaches with other weights give 1.107 (weights are Π_*t*_ *w*_*t*_), 1.111 (weights are *w*_*t*_ */w*_*t0*_), and 1.369 (weights are stabilized, i.e., Π_*t*_(*w*_*t*_ */w*_*t0*_)). We believe that the estimate in Table 4 is realistic because the fraction of significant differences among the variables predicting treatment choice (not shown) is 2/31 (both for comorbidity index), while these fractions for other three methods are 9/31, 17/31, and 21/31, respectively.

The outcomes and predictors in MSM are evaluated in each time point and considered separate observations. We use four time points (0, 2, 4, and 6 months after diagnoses) and, therefore, the dataset for MSM has the number of records four times larger than the number of patients. The observations from the same patient are not independent, therefore the GEE approach with a working matrix describing the correlation between time points of the same patient is used. The results presented in Table 4 are obtained using so-called 3-dependent working matrix in which the diagonal parameters (i.e., matrix elements on the three diagonals *W*_*i*, *i*+*j*_ ≡ *W*_*j*_, *j* = 1, 2, 3) are the same (among all rows represented by index *i*) and are subject for estimation. For majority of weighted and non-weighted stage-specific analyses the parameters were approximately estimated as *W*_1_ ≈ 0.7, *W*_2_ ≈ 0.4, and *W*_3_ ≈ 0.1. Also independent, exchangeable, autoregression, and unspecified working matrixes were tested. The estimates using these working matrixes and statistical criteria (such as the quasi-likelihood information criterion (QIC) by Pan [16] confirm the choice of 3-dependent matrix as optimal.

### Sensitivity Analysis

The above comparison of the estimates for time-independent and time-dependent treatments, for alternative approaches to calculate weights, and for different models of working correlation matrices in GEE is the first stage of our sensitivity analyses designed to assess the robustness of our findings and identify uncertainties of applying MSM to SEER-Medicare data. Other model specifications were tested in additional sensitivity studies allowing for assessment of the impact of assignment of the date of diagnosis and specific choice of point of time for treatment evaluation. We did not find substantial changes in the results varying respective model assumptions except the case when different numbers of time points were used. The removal of the last time point from the analysis (i.e., time point at 6th month after the date of diagnosis) results in more beneficial effects of all therapies. This observation suggests that delays of this length after diagnosis results in poorer survival.

The most important limitation of the above model is the assumed lack of unmeasured covariates related to treatment assignment and subsequent survival. Evidently, unmeasured tumor statuses (i.e., the triad of the T-, N-, and M-statuses) before and after treatment are such variables. They are measured at baseline only. To check the effect of this assumption we modeled dynamics of tumor statuses using stage-specific transition probabilities. First, distributions of T and N statuses at the time of diagnoses were used to randomly replace unknown statuses at baseline. Second, we modeled two-month stage-specific probabilities of increase of the statuses by 1 or 2 units, e.g., we used 5% for *T*_*n*_ → *T*_*n*+2_ and 20% for *T*_*n*_ → *T*_*n*+1_ for all stages and 5, 10, 25, and 25% for *M*_0_ → *M*_1_ for stages I, II, IIIA, and IIIB, respectively. Third, we modeled the effects of each treatment. Surgery and radiation therapy resulted to *T*_0_ and *N*_0_ with certain stage specific probabilities of subsequent recurrent tumor growth in two months, e.g., probabilities for *N*_0_ → *N*_1_ were 2, 10, 15, 15, and 15% for the five considered stages. Chemotherapy was assumed to have certain probability to improve *T*- and *N*-statuses (e.g., 50% for *T*_*n*_ → *T*_*n*-1_, 25% *T*_*n*_ → *T*_*n*_, and 25% *T*_*n*_ → *T*_*n*+1_) and to decrease the probability of metastasizing. We modeled TNM-statuses for all patients using these assumptions and then added these new variables to the MSM models. We detected the change in estimates (e.g., ORs for treatments without surgery of patients staged IIIA were 0.868 (instead of 0.920) for chemo- and radio-therapy, 0.711 (0.680) for chemotherapy and 0.769 (0.799) for radiotherapy), which however allowed our conclusions to remain the same. A minor change in parameter estimates occurred because information about patient death was not used, considering that probability of patient death strongly correlates with changes of TN- and especially of M-statuses. When we added this information into our modeling strategy (e.g., we assumed that probability of transition *M*_0_ → *M*_1_ depends on time to death as exp(*α*(*t*_*d*_ *—t*)), where *t* and *t*_*d*_ are the current time and time of diagnosis, we observed much higher changes in parameter estimates. However, estimating how realistic these results are is difficult, because it is challenging to distinguish between actual dependence of survival on *M*_1_ or that induced by our simulation strategy.

## Discussion

In this paper, we applied a set of statistical models to evaluate the stage-specific causal effects of surgical resection, radiation, and chemotherapy on survival of lung cancer patients, considering both time-independent and time-dependent treatments. Within the approaches for time-independent treatment, the treatment was represented by an eight-stratum variable that did not consider order of treatment. In the approach for time-dependent treatment the treatment was represented by a set of these variables reflecting treatments in all predetermined time points. Application of these causal inference methods allowed us to comprehensively evaluate the impact of lung cancer treatment while considering specific patient details regarding not only cancer characteristics but also specific comorbid states. Applying these novel methods to datasets that allow assembly of large cohorts of patients with relatively uncommon substages of lung cancer has the potential to provide data that can help close a current significant gap in evidence regarding lung cancer treatment effectiveness and patients survival, that is how to choose the optimal treatment for an older patient with lung cancer when considering not only details about their cancer but also the patients’ comorbid conditions. These methods can be used to assist development and enhancement of comprehensive guidelines for lung cancer treatment of older adults by considering the impact of dynamically changing comorbidities. By allowing cancer care providers and patients to make treatment decisions based on quantitative data and not simply subjective estimation of the impact of comorbid conditions, care of lung cancer patients can be more evidence-driven and better standardized. Considering the current prevalence of lung cancer in conjunction with the aging population, the application of these methods of causal inference to SEER-Medicare claims data, which had not previously been described, has the potential to significantly impact and improve patient care. Both methodological and substantive aspects of our approach are discussed in detail below.

### Methodological issues of modeling time-independent treatment

The idea of time-independent treatments reflects the point of view that the complete treatment is largely planned at the time of cancer diagnoses and then only minor changes are possible. In this approach cancer stage and patient characteristics at baseline dictate which modality or combinations of modalities define treatment options for patients. In total, eight possible time-independent treatments were considered: i.e., from “no treatment” option to the modes when all three treatments are applied.

Three issues are needed to keep in mind when interpreting the results with time-independent treatments. The first is that in practice the treatments are corrected during the treatment course because of multiple reasons. We discuss them below considering time-dependent treatments. For time-independent treatments we have only the resulting treatment patterns and they are not always resulted from decision at baseline. The second is that we need to make simplifying assumptions to limit the complexity of the analysis. For example, we consider only whether patients had surgery or not as part of their treatment, but in reality surgery *per se* can be of at least five different types depending on the extent of resection, such as pneumonectomy, lobar and sublobar resection, local treatment, and other (unknown extent). In this analysis we ignore the differences in the specific surgeries applied for the selected lung cancer patients. The third is that the treatments within a certain treatment pattern are still quite heterogeneous. Except for different types of the surgery, we also can deal with multiple surgeries of different types, and with different doses of chemo- and radio-therapy. Moreover, the patterns with different order (i.e., initial treatment and following courses) of treatments were not considered in this study as different treatments.

Selection bias is addressed through the method of pseudorandomization. The underlying idea of this approach is to assign a weight for each patient to create a situation where all patient characteristics for treatment-specific groups become similar. An important step in propensity-score-based analyses is testing the quality of pseudorandomization. Within the approach based on IPW, this testing can be performed formally, e.g., ANOVA test for continuous and *χ* ^2^ test for categorical predictors. Table 2 and S1 and S2 Tables represented the results of these testing for time-independent treatments and showed that *p*-values demonstrating strong significance for original patients groups (i.e., non-weighted) became non-significant when the weights were applied. This finding occurred for all variables predicting the treatment choice in the treatment model, indicating that this approach simulates “clinical trials” in which we select similar (randomized) groups for all variables except the treatment of interest. Treatment is the main predictor in the outcome model that is then estimated for weighted and non-weighted (for comparison) patient groups. The variables contributing to the treatment model (and, which, therefore, are not different for weighted population) can also be added to the outcome model as predictors. They are not expected to impact the treatment effect (because of lack of the correlation between them and treatment for weighted population) but they can have their own contribution to probability of survival. One additional conclusion from the analysis of time-independent treatment (Table 3) is that pseudorandomization did not strongly change the estimates for treatment effects in stratum-specific groups, suggesting that the selection bias is not critical or/and that the patient group in strata defined by a lung cancer stage is not very heterogeneous. However it can also mean that the list of predictors that impact treatment choice is not complete and that unmeasured variables important to treatment selection are not being considered. Examples of potentially important variables that were not available in the dataset and, therefore, were not considered in this study include overall functional status and specific pulmonary function measurements.

### Methodological issues of modeling time-dependent treatment

The assumptions of the models for time-independent treatments could be relaxed if additional longitudinal information available in data was used, such as the dates of specific treatments administered and changes in comorbidity patterns during individual treatment course. The comorbidity time patterns and treatment dates are reconstructed using ICD-9 procedure, CPT/HCPCS, and revenue center codes [12,14], which are all available in Medicare records. Availability of this longitudinal information allowed us to apply the MSM for Medicare data on lung cancer patients. In MSM we consider treatments taken at a certain time point of individual follow-up. All other time-dependent variables (such as disease indicators and comorbidity index) are also defined and evaluated in these predetermined times points. The treatment model in MSM is defined for each time point. Predicted probabilities of time-specific treatments are used to evaluate weights for pseudorandomization at each time point. The effects of specific treatments on the outcome are represented by a single treatment-specific measure resulting from some “averaging” over time-specific treatment effects. This “averaging” is effectively obtained within an outcome model (specifically a logistic model for weighted repeated measures model with generalized estimating equations) in which individual time-specific records (predictors and outcome) are considered correlated and this correlation is represented by an appropriate working matrix. Two important methodological steps have to be performed to support the results of the modeling: checking the quality of created pseudorandomization in all time points and performing a detailed sensitivity study.

Methodologically, addressing the selection bias for time-independent treatment is a routine procedure that does not provide researchers with flexibility if the counterfactual treatments and all predictors of the treatment and outcome are defined. In the case of MSM, the model can be defined and specified in several ways. The available flexibility in model specification is used to tune the model for a specific substantive case. The structure of the data and the nature of underlying substantive process in this present study differ from those for which MSM is typically applied. Usually, MSM is applied for a situation representing a steady state or data that can be considered as a stationary or almost stationary time series, where the researcher can incorporate the ideas of analyses of time series when different individual measurements are simply different measurements of the same steady-state. In the case of lung cancer, different time points cannot be considered as steady-state and respective MSM models therefore have to be tuned accordingly, which is why we made four modifications to the standard MSM formulation for binary outcomes ([7,8], a specific implementation for continuous outcomes are given in Faries et al. [17]). The first change is that we do not use stabilized weights but instead simply the usual IP weight that is a part of the stabilized weight. At baseline, the stabilized weights go to unit and do not provide the appropriate pseudorandomization for *t* = 0. The second change is that we do not multiply weights calculated for different time points of the same individual, because in our applications the quality of pseudorandomization was much better in the case when we used only the weight taken in the current time point. The third change is that we do not use the censoring component for MSM weights. We do not have censoring events in the follow-up period because of our strategy for patient selection. The fourth change is that we do preselect patients based on their previous treatment for the treatment model, but use the previous treatment as a predictor of current treatment.

The results of respective effects for time-dependent and time-independent treatments are qualitatively similar, but strongly deviate quantitatively (Table 4). The reason for this deviation is the contributions of time periods when no treatments were administered. For patients with time-independent pattern “no treatment,” these time periods contribute to the reference group both for time-dependent and time-independent treatments. However, they contribute differently for patients with other treatment patterns. In the case of MSM analysis of time-dependent treatments, time periods without any treatment are the referent group, while for the IP analysis of time-independent treatments they (being a part of an entire time-independent treatment pattern) are not a part of the no-treatment referent group. As a result, the referent group for time-dependent treatment has better survival. Many other opportunities for model specifications allowed us to perform detailed sensitivity studies. We found that there are not many effects due to pure technical issues such as different forms of the working matrix. The two detected sensitivities were due to substantive reasons. First, we found that our result was dependent on the time of the last point where we evaluated treatment and other predictors and predicted survival which in accordance with the idea discussed above that the outcome is not a stationary series during the individual treatment course. The survival probability (as well as death rate) is different for different time points after the date of diagnosis (Fig 1).

The second type of sensitivity analysis was aimed at addressing the most important limitation of the MSM, which is the assumed lack of unmeasured covariates related to treatment assignment and subsequent survival. Clearly, unmeasured tumor status before and after treatment is such a variable. We modeled dynamics of TNM-statuses and implemented their effects into the model. We found that unmeasured TNM-statuses can impact the estimates of treatment effects in MSM. One promising approach to deal with this issue is based on the stochastic process model [18] in which survival is modeled as a function of both observed (treatment and comorbidity) and unobserved (TNM-statuses) time-dependent covariates. The dynamic changes of these covariates can be modeled by stochastic differential equation(s) based on measurements for observed covariates and knowledge from clinical practice for unobserved TNM-statuses.

The analysis presented in this paper is based on MSM that currently is a standard approach in causal inference for longitudinal data. The use of this approach to estimate treatment effect with SEER-Medicare data, which is a very comprehensive dataset with details on cancer diagnosis linked to Medicare records, has not been previously described. This step in analysis of time-dependent treatment effect is necessary and methodologically unavoidable. This analysis also creates a basis for further studies of treatment effect involving some substantive (and, maybe, parametric) assumptions about dependences of mortality risks and treatment selection on the history of previous treatment and current comorbidities.

### Discussion of substantive results

The results obtained from our study can have direct practical applications. Specifically, the results can be used to help optimize the treatment decision for older patients with lung cancer both to help choose treatment that will provide the highest chance of survival while avoiding therapy that does not help or even have negative impact on patient’s survival. Choosing treatment for elderly patients with lung cancer is currently a common and critically important situation, considering that nearly 40% of all new lung cancer cases diagnosed are in patients aged 70+ years old [19]. Importantly, the use of population-based dataset provides the results that are generalizable to typical patients seen in very common clinical situations. Choosing therapy for an individual lung cancer patient is also an inherently complex process. While the stage of lung cancer at diagnosis is the most significant predictor of survival, other factors such as age, gender, race, tumor size and histology [20,21], SES, and comorbidities [22–26] also impact the treatment choice and effectiveness. Our current study also found that a better prognosis for survival was associated with younger age, smaller tumor size, higher SES, and fewer comorbidities, which is consistent with multiple other studies of these factors. However, this knowledge of factors important to prognosis has not necessarily been able to be translated into change in clinical practice to improve outcomes. Developing a tool that allows accurate estimates of the benefits of specific treatments for patients based on specific characteristics has the potential to assist the treatment decision process for individual patients. The results of this population-based study can allow clinicians to make the evidence-based treatment decisions, rather than having to rely on their subjective judgment. Perhaps most importantly, clinicians can make their objective decisions regarding whether the benefits of treatment outweigh the risks, rather than choosing treatment based on whether the patient is considered “too old” or “too sick” to tolerate and derive benefit from a treatment.

Age still appears to be a major factor influencing treatment choice in elderly patients [27–29]. However, treatment decisions for older patients should take into account many factors, including the benefits in life expectancy, treatment tolerance, and presence of and complications due to comorbidities. Unfortunately, information available on benefits and tolerability of different treatments in older adults is sparse, and the risk versus benefit has not been studied adequately. For example, the role of surgery in the elderly with lung cancer has significantly changed during the last 20 years due to advances in anesthetic management and surgical techniques which have allowed the inclusion of increasing numbers of the elderly patients in surgical studies. While some studies support an association between increased age and occurrence of postoperative complications [30], others do not [31,32]. In addition, surgical advances such as minimally invasive surgical techniques have improved the safety of surgery in elderly patients, and lung cancer surgery has been shown to be able to be safely performed with good long-term survival in extremely elderly patients such as octogenarians [33,34]. In our study, the beneficial effect of surgery on lung cancer patients’ survival decreased with advancing cancer stage. Elderly patients also receive fewer chemotherapy doses when compared with younger counterparts; however, they seem to derive the same benefit from adjuvant chemotherapy as younger patients do, with no significant increase in toxicity [35]. In our study, we observed that the beneficial effects of chemo-and also radiotherapy on survival increased with advancing lung cancer stage. In the literature, role of radiotherapy in aged patients with lung cancer also remains uncertain because of limited data: for example, some reports have shown that stereotactic body radiation is a safe and effective method of treating lung cancer in medically inoperable patients [36,37]. However, these retrospective studies are based on highly selected patients and their extrapolation to the general elderly population should be made with caution. Finally, even considering that the effectiveness, toxicity, and complications of different therapies change in humans with advancing age due to physiological aging-related changes, the presence and severity of comorbid conditions that generally are known to increase the risk of treatment toxicity make the lung cancer treatment decision process even more complex in elderly patients [5].

Unfortunately, existing guidelines do not provide detailed information that will help physician to make difficult decisions on treatment choice in older patients with lung cancer using specific patient characteristics [6]. In addition, the majority of recommendations are made based on retrospective data, which potentially are limited by selection bias that can make it difficult to determine if the results can be generalized to be applicable to a specific patient [35]. Importantly, these decisions cannot be done on the basis of chronological age alone but must incorporate all characteristics of patients, including specific tumor details as well as their other medical comorbidities. Attempts to provide informative tools that assist treatment decisions have been made: e.g., a comprehensive geriatric assessment (CGA) has been suggested to estimate a patient’s functional status, the presence of comorbidities, mental status and emotional conditions, nutritional status, polypharmacy and the presence or absence of geriatric syndromes [38]. However, even when the International Society of Geriatric Oncology recommended a CGA-based approach to elderly cancer patients [39], this method has not been prospectively validated as a prognostic and predictive factor for treatment-related toxicity and outcome. The methods described in our study have the potentially to much more significantly be integrated into clinical care. The application of the standard approach of propensity scoring that is broadly used in analyses of treatment effects using administrative data [40,41]) to SEER-Medicare data in this study allowed the development of statistical methodologies that can be implemented into a new computational tool for physicians. This tool can be used by clinicians in choosing the optimal lung cancer treatment that balances treatment benefits and risks for the patient based on individual lung cancer patient’s characteristics.

## Conclusion

In summary, we investigated in this study how applying methods of causal inference to large scale observational data such as SEER-Medicare can help clarify the effects of different treatment modalities on lung cancer survival. Specifically, we estimated causal treatment effects on overall survival of lung cancer patients for counterfactual time-independent and time-dependent treatment modalities. We also tested the plausibility of the model assumptions with sensitivity studies. This study provides new information on the causal effects of different treatment modalities on overall survival in lung cancer patients, as well as the effects of socioeconomic factors, tumor characteristics, age groups, and baseline/dynamic comorbidities on treatment choice and overall survival in lung cancer patients. The results also confirm that Medicare data is a powerful source for evaluating causal effects of time-dependent treatment in older adult patients with cancer diagnosis.

## Supporting Information

S1 Table The numbers of patients in treatment groups and percentage calculated without the IP weights and p-values of the *χ* ^2^-tests for treatment-group comparison of stage-specific cohorts of lung cancer patients calculated for original populations. (PDF)

S2 Table The numbers of patients in treatment groups and percentage calculated with the IP weights and p-values of the *χ* ^2^-tests for treatment-group comparison of stage-specific cohorts of lung cancer patients calculated for pseudorandomized populations. (PDF)

S3 Table The p-values of the *χ* ^2^-tests for treatment-group comparison of stage-specific cohorts of lung cancer patients calculated for original and pseudorandomized (i.e., weighted) populations in specific times after diagnosis. (PDF)
