# Supplementary material for: Causal Effects of Time-Dependent Treatments in Older Patients with Non-Small Cell Lung Cancer
Source: PLoS One. 2015 Apr 7;10(4):e0121406. doi: 10.1371/journal.pone.0121406 (PMC4388569; doi:10.1371/journal.pone.0121406)
Supplement: S2 Table — (PDF) [file pone.0121406.s002.pdf]

Supplementary Table S2. The numbers of patients in treatment groups and percentage calculated with the IP weights and p-values of the χ^2-tests for treatment-group comparison of stage-specific cohorts of lung cancer patients calculated for pseudorandomized populations.

Stage	Variable	No_Treatment	Che	Rad	Che_Rad	Sur	Sur_Che	Sur_Rad	Sur_Che_Rad	p_value
I	Sex									
I	Male	2580 (51.83)	451 (52.18)	2012 (49.73)	1406 (50.55)	8617 (50.69)	646 (50.30)	649 (51.10)	329 (50.47)	0.9877
I	Female	2597 (48.17)	353 (47.82)	1967 (50.27)	1140 (49.45)	8798 (49.31)	543 (49.70)	579 (48.90)	228 (49.53)	
I	Race									
I	White	4680 (93.16)	716 (93.24)	3691 (93.05)	2287 (93.77)	16590 (93.52)	1125 (91.49)	1160 (93.52)	518 (92.34)	0.8535
I	Non-White	497 (6.84)	88 (6.76)	288 (6.95)	259 (6.23)	825 (6.48)	64 (8.51)	68 (6.48)	39 (7.66)	
I	Age (years)									
I	65-69	654 (22.89)	156 (20.88)	480 (19.21)	471 (18.38)	3976 (19.77)	358 (19.45)	290 (19.22)	186 (19.56)	0.9945
I	70-74	1028 (28.63)	227 (28.82)	879 (28.22)	715 (28.73)	5607 (29.18)	438 (28.98)	410 (28.30)	205 (27.09)	
I	75-79	1294 (26.32)	230 (25.75)	1050 (27.38)	731 (28.21)	4852 (27.37)	296 (26.57)	316 (27.39)	110 (28.29)	
I	80-84	1235 (15.30)	144 (16.57)	976 (17.15)	446 (16.51)	2337 (16.18)	80 (17.90)	170 (17.36)	45 (15.17)	
I	85+	966 (6.86)	47 (7.98)	594 (8.03)	183 (8.17)	643 (7.49)	17 (7.10)	42 (7.72)	11 (9.88)	
I	T-Status									
I	T1	1934 (46.35)	191 (44.37)	1600 (43.85)	599 (44.54)	9279 (44.60)	362 (48.05)	440 (44.83)	153 (43.96)	n/a
I	T2	2155 (44.38)	348 (45.58)	1598 (45.78)	1337 (45.20)	7683 (45.52)	799 (41.06)	710 (45.19)	351 (41.27)	
I<br>I	T3<br>TX	1088 (9.27)	265 (10.06)	781 (10.37)	610 (10.26)	453 (9.88)	28 (10.90)	78 (9.98)	53 (14.77)	
I	SES (% black)									
I	0	1676 (32.15)	240 (34.62)	1323 (32.74)	781 (33.56)	5917 (33.43)	407 (30.35)	417 (33.89)	206 (32.81)	0.9898
I	1	1640 (35.88)	279 (33.61)	1262 (34.70)	775 (34.93)	6350 (34.52)	410 (36.36)	425 (34.65)	191 (36.04)	
I	2	1861 (31.96)	285 (31.77)	1394 (32.55)	990 (31.50)	5148 (32.05)	372 (33.29)	386 (31.47)	160 (31.15)	
I	SES (% college)									
I	0	1933 (29.80)	284 (33.32)	1386 (32.96)	1024 (31.78)	4839 (31.58)	334 (35.05)	383 (33.17)	180 (34.16)	0.6077
I	1	1691 (31.63)	253 (34.47)	1348 (32.81)	801 (33.70)	5702 (32.85)	379 (30.41)	376 (31.59)	179 (31.74)	
I	2	1553 (38.57)	267 (32.21)	1245 (34.23)	721 (34.51)	6874 (35.57)	476 (34.53)	469 (35.24)	198 (34.10)	
I	SES (% poverty)									
I	0	1485 (37.48)	291 (37.25)	1287 (35.80)	774 (34.78)	6830 (36.43)	490 (34.36)	452 (35.04)	195 (32.62)	0.3820
I	1	1719 (32.95)	237 (31.42)	1346 (32.22)	789 (34.66)	5872 (33.52)	388 (33.00)	412 (32.11)	174 (30.62)	
I	2	1973 (29.57)	276 (31.33)	1346 (31.98)	983 (30.55)	4713 (30.05)	311 (32.63)	364 (32.85)	188 (36.75)	

I	Histology									
I	AC	1429 (45.12)	274 (37.65)	1093 (41.01)	635 (42.92)	9154 (42.43)	599 (40.67)	547 (42.31)	217 (43.91)	0.4553
I	SCC	1308 (31.00)	239 (35.53)	1481 (32.36)	1027 (31.45)	5371 (31.25)	344 (33.30)	447 (31.63)	205 (33.20)	
I	Other	2440 (23.88)	291 (26.83)	1405 (26.63)	884 (25.63)	2890 (26.33)	246 (26.02)	234 (26.06)	135 (22.89)	
I	Comorbidity									
I	0	918 (38.88)	201 (35.88)	994 (33.87)	636 (34.17)	7967 (36.59)	586 (34.35)	469 (37.92)	216 (34.51)	0.4361
I	1	1147 (26.35)	201 (24.49)	1119 (26.54)	645 (26.77)	4606 (25.95)	313 (27.08)	363 (25.98)	149 (25.93)	
I	2	1133 (17.64)	190 (19.92)	962 (19.52)	570 (19.61)	2826 (18.81)	165 (20.80)	229 (18.16)	117 (16.73)	
I	3	1079 (11.28)	149 (12.38)	615 (12.88)	443 (12.65)	1492 (12.78)	95 (11.12)	118 (12.54)	48 (12.29)	
I	4	900 (5.85)	63 (7.33)	289 (7.20)	252 (6.80)	524 (5.88)	30 (6.65)	49 (5.39)	27 (10.55)	
II	Sex									
II	Male	256 (61.37)	77 (51.87)	245 (57.68)	390 (56.54)	1040 (57.08)	311 (57.13)	444 (55.79)	309 (58.61)	0.6365
II	Female	229 (38.63)	60 (48.13)	203 (42.32)	279 (43.46)	799 (42.92)	276 (42.87)	340 (44.21)	222 (41.39)	
II	Race									
II	White	434 (93.49)	137 (100.0)	411 (94.85)	610 (94.25)	1766 (93.78)	560 (94.28)	750 (95.08)	502 (93.48)	0.9867
II	Non-White	51 (6.51)		37 (5.15)	59 (5.75)	73 (6.22)	27 (5.72)	34 (4.92)	29 (6.52)	
II	Age (years)									
II	65-69	83 (24.30)	27 (24.58)	63 (21.20)	150 (26.37)	384 (24.02)	202 (22.78)	214 (24.44)	192 (22.71)	0.8566
II	70-74	107 (34.24)	43 (27.27)	108 (31.39)	217 (30.28)	569 (32.08)	198 (27.59)	281 (29.67)	187 (34.25)	
II	75-79	118 (23.46)	42 (27.06)	129 (28.81)	189 (26.07)	519 (26.39)	140 (26.73)	189 (27.13)	111 (24.52)	
II	80-84	104 (13.87)	14 (15.99)	94 (13.59)	91 (11.99)	294 (13.27)	41 (22.91)	81 (14.51)	41 (18.53)	
II	85+	73 (4.14)	11 (5.10)	54 (5.01)	22 (5.30)	73 (4.24)		19 (4.24)		
II	T-Status									
II	T1	112 (26.30)	29 (25.98)	88 (22.29)	115 (26.11)	538 (24.90)	157 (23.67)	207 (25.56)	123 (25.41)	0.9932
II	T2	231 (55.88)	62 (55.72)	214 (60.68)	300 (56.10)	1120 (57.65)	378 (54.27)	520 (57.94)	324 (55.74)	
II	T3	94 (12.13)	26 (12.42)	72 (11.07)	150 (11.66)	134 (11.13)	52 (22.06)	42 (11.29)	69 (10.61)	
II	TX	48 (5.69)	20 (5.88)	74 (5.97)	104 (6.13)	47 (6.32)		15 (5.21)	15 (8.25)	
II	SES (% black)									
II	0	145 (33.29)	41 (36.32)	153 (37.36)	206 (33.85)	661 (33.87)	194 (33.97)	300 (35.68)	180 (33.85)	0.9999
II	1	155 (35.17)	43 (31.87)	152 (32.33)	198 (33.90)	655 (34.06)	208 (33.82)	274 (33.83)	189 (32.87)	
II	2	185 (31.53)	53 (31.81)	143 (30.30)	265 (32.25)	523 (32.07)	185 (32.20)	210 (30.49)	162 (33.29)	
II	SES (% college)									
II	0	177 (27.86)	43 (33.89)	161 (28.68)	241 (30.78)	511 (29.98)	146 (31.07)	257 (31.12)	158 (33.14)	0.9938
II	1	152 (33.90)	44 (32.33)	162 (31.94)	216 (33.73)	581 (32.91)	190 (32.28)	257 (31.51)	175 (31.17)	

II	2	156 (38.24)	50 (33.78)	125 (39.38)	212 (35.50)	747 (37.11)	251 (36.65)	270 (37.36)	198 (35.70)	
II	SES (% poverty)									
II	0	133 (36.09)	47 (34.48)	162 (41.87)	249 (36.47)	679 (37.07)	216 (37.48)	310 (37.76)	237 (34.99)	0.8949
II	1	164 (35.95)	41 (30.42)	155 (30.97)	198 (34.35)	654 (33.93)	205 (31.57)	250 (32.44)	159 (35.41)	
II	2	188 (27.96)	49 (35.10)	131 (27.16)	222 (29.18)	506 (29.00)	166 (30.96)	224 (29.80)	135 (29.61)	
II	Histology									
II	AC	132 (43.18)	41 (36.81)	97 (40.39)	154 (39.67)	874 (40.23)	295 (43.62)	376 (40.40)	256 (39.70)	0.9919
II	SCC	144 (35.24)	50 (38.41)	193 (35.04)	291 (34.53)	638 (35.41)	168 (33.14)	274 (35.31)	168 (35.79)	
II	Other	209 (21.58)	46 (24.79)	158 (24.57)	224 (25.79)	327 (24.36)	124 (23.24)	134 (24.29)	107 (24.51)	
II	Comorbidity									
II	0	81 (37.15)	36 (28.17)	110 (33.18)	194 (34.70)	671 (33.02)	290 (30.87)	264 (34.32)	205 (31.90)	0.9788
II	1	119 (27.80)	42 (28.42)	140 (24.64)	159 (26.44)	481 (26.73)	138 (32.42)	243 (26.37)	154 (27.74)	
II	2	106 (18.51)	23 (23.74)	99 (22.65)	150 (20.76)	374 (20.63)	95 (19.45)	161 (21.83)	97 (19.88)	
II	3	101 (11.36)	36 (19.66)	66 (13.68)	97 (12.10)	211 (12.72)	44 (12.51)	86 (12.23)	63 (12.19)	
II	4	78 (5.18)		33 (5.84)	69 (6.00)	102 (6.91)	20 (4.75)	30 (5.25)	12 (8.30)	
IIIA	Sex									
IIIA	Male	1409 (58.77)	553 (57.07)	1425 (56.54)	2598 (56.91)	697 (58.06)	252 (59.06)	477 (57.77)	599 (58.67)	0.9926
IIIA	Female	1122 (41.23)	394 (42.93)	1069 (43.46)	1857 (43.09)	595 (41.94)	231 (40.94)	401 (42.23)	416 (41.33)	
IIIA	Race									
IIIA	White	2271 (91.79)	883 (92.14)	2310 (91.88)	4052 (91.92)	1209 (92.29)	450 (94.56)	835 (93.05)	967 (91.04)	0.5864
IIIA	Non-White	260 (8.21)	64 (7.86)	184 (8.12)	403 (8.08)	83 (7.71)	33 (5.44)	43 (6.95)	48 (8.96)	
IIIA	Age (years)									
IIIA	65-69	328 (20.74)	189 (21.71)	327 (21.05)	1086 (21.02)	271 (22.36)	151 (22.98)	236 (20.75)	368 (21.43)	0.9771
IIIA	70-74	535 (30.74)	294 (28.63)	587 (28.67)	1440 (28.78)	380 (29.76)	186 (27.61)	299 (30.71)	360 (30.00)	
IIIA	75-79	644 (25.78)	264 (26.35)	686 (26.39)	1169 (26.33)	384 (27.16)	100 (24.19)	244 (27.53)	222 (26.43)	
IIIA	80-84	606 (15.62)	160 (15.88)	573 (16.62)	584 (16.13)	201 (14.72)	46 (25.22)	77 (14.25)	65 (22.15)	
IIIA	85+	418 (7.13)	40 (7.43)	321 (7.26)	176 (7.73)	56 (6.00)		22 (6.77)		
IIIA	T-Status									
IIIA	T1	422 (16.51)	160 (16.52)	359 (15.83)	675 (16.42)	244 (16.81)	121 (16.38)	156 (15.64)	183 (15.82)	0.9783
IIIA	T2	936 (38.36)	385 (39.18)	893 (38.61)	1740 (38.41)	471 (38.41)	249 (40.67)	335 (37.78)	464 (39.11)	
IIIA	T3	480 (25.30)	161 (24.70)	612 (25.01)	987 (25.06)	530 (25.49)	88 (28.87)	362 (25.72)	275 (27.50)	
IIIA	TX	693 (19.83)	241 (19.60)	630 (20.56)	1053 (20.11)	47 (19.29)	25 (14.09)	25 (20.85)	93 (17.57)	
IIIA	SES (% black)									
IIIA	0	819 (33.46)	329 (34.78)	882 (34.55)	1507 (33.84)	423 (33.32)	172 (35.62)	331 (28.76)	345 (34.63)	0.9523

IIIA	1	803 (33.19)	331 (32.26)	812 (33.00)	1449 (33.62)	467 (33.32)	178 (33.30)	293 (35.41)	339 (31.19)	
IIIA	2	909 (33.36)	287 (32.96)	800 (32.44)	1499 (32.53)	402 (33.36)	133 (31.08)	254 (35.83)	331 (34.18)	
IIIA	SES (% college)									
IIIA	0	903 (32.83)	309 (32.31)	834 (33.10)	1511 (33.01)	405 (33.22)	101 (34.68)	259 (32.15)	293 (34.99)	1.0000
IIIA	1	880 (32.83)	330 (33.24)	885 (33.62)	1466 (33.23)	406 (32.51)	165 (32.06)	283 (33.93)	301 (30.92)	
IIIA	2	748 (34.34)	308 (34.45)	775 (33.28)	1478 (33.76)	481 (34.27)	217 (33.26)	336 (33.92)	421 (34.09)	
IIIA	SES (% poverty)									
IIIA	0	750 (35.36)	329 (34.86)	798 (34.53)	1525 (34.35)	447 (34.75)	220 (32.68)	353 (33.11)	409 (33.66)	0.7707
IIIA	1	810 (32.24)	318 (32.51)	841 (32.94)	1481 (32.93)	453 (34.79)	142 (38.79)	299 (32.93)	327 (30.68)	
IIIA	2	971 (32.39)	300 (32.64)	855 (32.53)	1449 (32.73)	392 (30.46)	121 (28.53)	226 (33.97)	279 (35.66)	
IIIA	Histology									
IIIA	AC	540 (32.37)	290 (30.81)	567 (30.44)	1154 (30.33)	587 (31.21)	252 (29.45)	401 (28.70)	471 (32.62)	0.9469
IIIA	SCC	717 (34.97)	296 (35.03)	1069 (34.58)	1666 (35.07)	459 (35.37)	121 (38.87)	299 (34.13)	315 (36.13)	
IIIA	Other	1274 (32.66)	361 (34.16)	858 (34.98)	1635 (34.60)	246 (33.43)	110 (31.68)	178 (37.17)	229 (31.25)	
IIIA	Comorbidity									
IIIA	0	348 (27.78)	253 (26.47)	549 (24.46)	1231 (26.28)	420 (25.76)	222 (23.94)	290 (25.60)	399 (25.52)	0.6010
IIIA	1	490 (25.50)	244 (25.42)	668 (26.26)	1127 (24.92)	355 (25.91)	128 (25.68)	268 (23.05)	291 (25.37)	
IIIA	2	562 (21.25)	211 (21.72)	617 (22.75)	1046 (22.32)	260 (22.50)	69 (19.94)	174 (24.16)	184 (21.99)	
IIIA	3	591 (16.14)	159 (16.34)	453 (16.75)	671 (16.59)	170 (16.19)	50 (24.52)	118 (15.19)	113 (18.21)	
IIIA	4	540 (9.34)	80 (10.05)	207 (9.79)	380 (9.88)	87 (9.63)	14 (5.92)	28 (12.00)	28 (8.91)	
IIIB	Sex									
IIIB	Male	4105 (56.35)	1914 (56.15)	2052 (55.82)	4143 (56.50)	611 (60.23)	203 (64.32)	244 (53.07)	344 (59.60)	0.3624
IIIB	Female	3834 (43.65)	1435 (43.85)	1487 (44.18)	2867 (43.50)	569 (39.77)	186 (35.68)	161 (46.93)	228 (40.40)	
IIIB	Race									
IIIB	White	7158 (90.59)	3089 (90.40)	3223 (90.49)	6273 (90.04)	1107 (84.49)	374 (94.07)	388 (91.32)	538 (92.05)	0.0737
IIIB	Non-White	781 (9.41)	260 (9.60)	316 (9.51)	737 (9.96)	73 (15.51)	15 (5.93)	17 (8.68)	34 (7.95)	
IIIB	Age (years)									
IIIB	65-69	860 (19.23)	670 (18.97)	554 (18.55)	1805 (18.63)	237 (20.43)	111 (19.50)	109 (16.49)	173 (17.38)	0.9985
IIIB	70-74	1502 (26.41)	1020 (26.65)	841 (27.08)	2267 (26.44)	367 (27.05)	140 (22.40)	132 (24.85)	217 (26.91)	
IIIB	75-79	1882 (25.02)	956 (24.92)	916 (25.53)	1711 (24.95)	342 (27.29)	92 (20.62)	96 (23.98)	127 (26.28)	
IIIB	80-84	1851 (17.95)	527 (18.52)	813 (17.94)	877 (18.22)	189 (15.54)	46 (37.48)	50 (18.77)	55 (29.43)	
IIIB	85+	1844 (11.39)	176 (10.94)	415 (10.90)	350 (11.76)	45 (9.69)		18 (15.90)		
IIIB	T-Status									
IIIB	T1	52 (1.26)	48 (1.68)	57 (1.38)	159 (1.39)	29 (6.50)	21 (5.95)	30 (5.19)	16 (4.22)	0.4964

IIIB	T2	105 (2.73)	77 (3.29)	80 (2.73)	368 (2.72)					
IIIB	T3	86 (2.79)	42 (2.56)	132 (2.43)	247 (2.47)				29 (2.25)	
IIIB	T4	4512 (60.95)	1870 (61.11)	2123 (61.17)	4081 (60.49)	1081 (55.25)	326 (49.86)	344 (66.05)	440 (65.24)	
IIIB	TX	3184 (32.27)	1312 (31.35)	1147 (32.29)	2155 (32.94)	70 (38.26)	42 (44.19)	31 (28.76)	87 (28.29)	
IIIB	SES (% black)									
IIIB	0	2587 (32.97)	1096 (32.59)	1194 (32.79)	2294 (32.58)	397 (31.30)	141 (33.93)	143 (33.93)	206 (33.06)	0.9310
IIIB	1	2571 (32.99)	1134 (32.57)	1184 (32.74)	2265 (33.10)	414 (31.33)	142 (37.88)	145 (31.60)	193 (27.98)	
IIIB	2	2781 (34.04)	1119 (34.84)	1161 (34.47)	2451 (34.32)	369 (37.37)	106 (28.19)	117 (34.47)	173 (38.97)	
IIIB	SES (% college)									
IIIB	0	2875 (34.99)	975 (33.91)	1288 (34.25)	2492 (34.86)	324 (38.78)	107 (34.26)	128 (33.31)	188 (33.96)	0.9799
IIIB	1	2550 (32.68)	1133 (33.25)	1200 (33.46)	2396 (33.18)	371 (29.77)	122 (38.78)	133 (35.59)	179 (33.36)	
IIIB	2	2514 (32.33)	1241 (32.83)	1051 (32.29)	2122 (31.96)	485 (31.45)	160 (26.97)	144 (31.10)	205 (32.68)	
IIIB	SES (% poverty)									
IIIB	0	2298 (31.81)	1222 (31.72)	1051 (31.88)	2273 (31.52)	439 (31.05)	168 (41.90)	142 (29.77)	224 (35.44)	0.3551
IIIB	1	2611 (33.01)	1114 (33.91)	1237 (33.28)	2286 (33.60)	413 (29.77)	124 (29.48)	151 (32.40)	189 (25.08)	
IIIB	2	3030 (35.17)	1013 (34.37)	1251 (34.84)	2451 (34.88)	328 (39.18)	97 (28.62)	112 (37.83)	159 (39.48)	
IIIB	Histology									
IIIB	AC	2958 (35.89)	1782 (35.67)	897 (36.10)	1988 (36.23)	594 (34.74)	219 (35.35)	152 (31.44)	190 (30.63)	0.9473
IIIB	SCC	1421 (28.63)	455 (27.93)	1437 (27.56)	2487 (28.13)	370 (27.21)	89 (32.99)	165 (26.54)	239 (30.19)	
IIIB	Other	3560 (35.48)	1112 (36.40)	1205 (36.34)	2535 (35.64)	216 (38.05)	81 (31.67)	88 (42.02)	143 (39.19)	
IIIB	Comorbidity									
IIIB	0	595 (18.06)	569 (18.09)	649 (17.15)	1539 (17.20)	428 (15.20)	167 (14.55)	125 (19.65)	193 (17.36)	0.2881
IIIB	1	1079 (20.27)	668 (20.52)	877 (20.45)	1625 (19.83)	301 (18.80)	83 (15.75)	104 (20.06)	145 (20.65)	
IIIB	2	1545 (21.41)	751 (21.09)	818 (21.34)	1593 (21.48)	209 (20.56)	69 (17.73)	98 (22.08)	120 (19.69)	
IIIB	3	2122 (21.02)	792 (21.56)	701 (21.74)	1329 (21.36)	150 (19.45)	41 (34.01)	54 (17.04)	76 (25.39)	
IIIB	4	2598 (19.24)	569 (18.73)	494 (19.32)	924 (20.13)	92 (25.98)	29 (17.96)	24 (21.17)	38 (16.92)	
IV	Sex									
IV	Male	8024 (55.33)	4898 (55.78)	3585 (55.21)	7852 (54.75)	327 (53.86)	185 (51.33)	143 (56.61)	252 (44.38)	0.1569
IV	Female	7451 (44.67)	3745 (44.22)	2876 (44.79)	5885 (45.25)	306 (46.14)	149 (48.67)	108 (43.39)	183 (55.62)	
IV	Race									
IV	White	14013 (91.43)	7998 (91.62)	5975 (91.46)	12516 (91.31)	589 (89.42)	322 (92.37)	239 (93.50)	397 (88.65)	0.8942
IV	Non-White	1462 (8.57)	645 (8.38)	486 (8.54)	1221 (8.69)	44 (10.58)	12 (7.63)	12 (6.50)	38 (11.35)	
IV	Age (years)									
IV	65-69	2287 (21.61)	2121 (21.51)	1226 (21.42)	3702 (21.22)	134 (23.78)	92 (20.24)	67 (23.77)	146 (18.23)	0.4444

IV	70-74	3395 (27.91)	2696 (28.17)	1744 (27.92)	4423 (27.80)	209 (29.35)	126 (24.56)	94 (26.86)	148 (25.99)	
IV	75-79	3948 (25.61)	2376 (25.77)	1745 (26.02)	3367 (25.66)	173 (22.96)	81 (20.58)	58 (24.10)	110 (23.31)	
IV	80-84	3379 (16.35)	1141 (16.17)	1182 (16.53)	1675 (16.42)	88 (13.97)	35 (34.61)	32 (25.27)	31 (32.47)	
IV	85+	2466 (8.52)	309 (8.38)	564 (8.11)	570 (8.91)	29 (9.94)				
IV	T-Status									
IV	T1	2177 (15.52)	1296 (15.53)	1055 (15.49)	2058 (15.51)	189 (12.85)	79 (21.54)	82 (16.94)	108 (19.57)	0.1920
IV	T2	4160 (31.34)	2318 (31.55)	2338 (31.25)	4963 (31.45)	229 (28.24)	120 (23.76)	114 (35.47)	183 (24.04)	
IV	T3	253 (1.97)	153 (1.89)	134 (2.02)	313 (1.94)	20 (2.53)	13 (1.26)	34 (21.69)	16 (1.70)	
IV	T4	2807 (16.06)	1710 (15.76)	707 (16.11)	1951 (16.15)	106 (15.52)	66 (20.64)		59 (18.34)	
IV	TX	6078 (35.11)	3166 (35.27)	2227 (35.14)	4452 (34.95)	89 (40.86)	56 (32.80)	31 (25.91)	69 (36.34)	
IV	SES (% black)									
IV	0	4962 (32.60)	2836 (32.69)	2202 (32.68)	4547 (32.46)	221 (30.12)	110 (32.96)	91 (31.03)	141 (24.69)	0.1989
IV	1	5035 (33.10)	2976 (33.49)	2124 (33.09)	4524 (33.08)	227 (36.40)	132 (30.95)	83 (28.82)	146 (28.74)	
IV	2	5478 (34.30)	2831 (33.82)	2135 (34.24)	4666 (34.46)	185 (33.49)	92 (36.09)	77 (40.15)	148 (46.57)	
IV	SES (% college)									
IV	0	5471 (32.92)	2577 (32.78)	2137 (32.94)	4398 (32.98)	198 (34.23)	77 (35.22)	71 (42.38)	146 (33.69)	0.4817
IV	1	5136 (33.28)	2862 (33.49)	2221 (33.54)	4582 (33.24)	189 (33.48)	107 (38.19)	94 (28.82)	136 (29.52)	
IV	2	4868 (33.80)	3204 (33.73)	2103 (33.52)	4757 (33.78)	246 (32.29)	150 (26.60)	86 (28.80)	153 (36.79)	
IV	SES (% poverty)									
IV	0	4679 (33.45)	3213 (33.63)	2056 (33.74)	4934 (33.44)	224 (33.17)	142 (29.64)	100 (30.50)	154 (28.06)	0.9934
IV	1	5129 (33.01)	2821 (33.03)	2233 (32.93)	4486 (33.07)	226 (33.95)	114 (35.41)	81 (36.82)	136 (37.41)	
IV	2	5667 (33.55)	2609 (33.34)	2172 (33.32)	4317 (33.49)	183 (32.88)	78 (34.95)	70 (32.68)	145 (34.53)	
IV	Histology									
IV	AC	5068 (37.95)	3858 (38.17)	2303 (37.64)	5261 (37.74)	330 (37.08)	202 (39.58)	127 (43.21)	194 (28.28)	0.0107
IV	SCC	2224 (18.31)	1313 (17.68)	1483 (17.86)	2771 (18.00)	144 (15.94)	46 (22.35)	60 (25.53)	108 (20.74)	
IV	Other	8183 (43.74)	3472 (44.15)	2675 (44.51)	5705 (44.25)	159 (46.97)	86 (38.07)	64 (31.26)	133 (50.98)	
IV	Comorbidity									
IV	0	1402 (14.29)	1601 (14.27)	912 (13.82)	2048 (13.87)	211 (12.22)	105 (10.64)	70 (19.73)	108 (11.27)	0.0773
IV	1	2052 (17.52)	1722 (17.57)	1366 (17.59)	2512 (17.40)	141 (15.20)	97 (17.96)	56 (20.10)	112 (15.22)	
IV	2	2717 (20.65)	1861 (20.86)	1539 (20.65)	3043 (20.48)	112 (18.84)	72 (20.76)	55 (25.48)	102 (17.72)	
IV	3	3751 (23.54)	1892 (23.47)	1520 (23.80)	3467 (23.72)	97 (24.93)	40 (23.39)	53 (21.74)	76 (31.57)	
IV	4	5553 (23.99)	1567 (23.82)	1124 (24.13)	2667 (24.53)	72 (28.80)	20 (27.25)	17 (12.95)	37 (24.22)	
