# Supplementary material for: Causal Effects of Time-Dependent Treatments in Older Patients with Non-Small Cell Lung Cancer
Source: PLoS One. 2015 Apr 7;10(4):e0121406. doi: 10.1371/journal.pone.0121406 (PMC4388569; doi:10.1371/journal.pone.0121406)
Supplement: S3 Table — (PDF) [file pone.0121406.s003.pdf]

Supplementary Table S3. The p-values of the χ^2 -tests for treatment-group comparison of stage-specific cohorts of lung cancer patients calculated for original and pseudorandomized (i.e., weighted) populations in specific times after diagnosis.

Time after diagnosis<br>Weight	0 mon<br>No	0 mon<br>IP	2 mon<br>No	2 mon<br>IP	4 mon<br>No	4 mon<br>IP	6 mon<br>No	6 mon<br>IP
Stage I								
Previous Treatment			<.0001	0.9978	<.0001	0.9970	<.0001	0.9896
Sex	<.0001	0.8713	<.0001	0.9496	0.0004	0.8187	0.1929	0.2033
Age	<.0001	1.0000	<.0001	0.9101	<.0001	0.5948	<.0001	0.9847
SES(black)	0.0387	0.6058	0.5138	0.4897	0.0452	0.9880	0.5985	0.9389
SES(college)	0.0032	0.9016	0.0095	0.5593	0.5534	0.9143	0.4979	0.0601
SES(poverty)	0.0011	0.8915	0.0006	0.9161	<.0001	0.7915	0.0735	0.5047
Histology	<.0001	0.9973	<.0001	0.6084	<.0001	0.3377	0.0013	0.9029
Comorbidity	<.0001	0.9999	<.0001	0.9378	0.0002	0.0419	<.0001	0.0080
Stage II								
Previous Treatment			<.0001	0.8928	<.0001	0.8925	<.0001	0.9996
Sex	0.0104	0.8049	0.1790	0.9885	0.7002	0.9063	0.2387	0.2378
Age	0.0145	0.9995	<.0001	0.9699	<.0001	0.9877	0.0004	0.9825
SES(black)	0.0462	0.9433	0.3824	0.9315	0.2240	0.2568	0.0095	0.9777
SES(college)	0.4023	0.9720	0.1015	0.7124	0.2682	0.2449	0.5769	0.6489
SES(poverty)	0.0043	0.9141	0.3922	0.9676	0.1078	0.1510	0.9320	0.8626
Histology	0.0178	0.9120	<.0001	0.9486	0.0661	0.6836	0.4648	0.9986
Comorbidity	0.0119	0.9985	0.0856	0.9562	0.6291	0.5455	0.0786	0.6887
Stage IIIA								
Previous Treatment			<.0001	0.8598	<.0001	0.9975	<.0001	0.9999
Sex	0.0011	0.6611	0.0008	0.7876	0.3700	0.9967	0.9970	0.8019
Age	<.0001	1.0000	<.0001	0.8278	<.0001	0.2204	<.0001	0.4654
SES(black)	0.0994	0.9968	0.0240	0.3729	0.8677	0.6210	0.5490	0.6749
SES(college)	0.0530	0.9965	0.0226	0.3592	0.2944	0.9069	0.1254	0.9490
SES(poverty)	0.0028	0.9959	0.0015	0.6073	0.0168	0.2734	0.0421	0.7734
Histology	<.0001	0.9997	<.0001	0.9411	<.0001	0.6941	0.0003	0.9828
Comorbidity	<.0001	0.9999	<.0001	0.3645	0.0001	0.5745	<.0001	0.4860
Stage IIIB								
Previous Treatment			<.0001	0.9880	<.0001	0.8684	<.0001	0.9970
Sex	<.0001	0.9847	<.0001	0.3328	<.0001	0.2997	0.0016	0.2894
Age	<.0001	0.9949	<.0001	0.8422	<.0001	0.5317	<.0001	0.5965
SES(black)	<.0001	0.9976	0.1897	0.7715	0.0571	0.7797	0.0887	0.8916
SES(college)	<.0001	0.9968	<.0001	0.5064	<.0001	0.9091	0.0006	0.5093
SES(poverty)	<.0001	0.9996	<.0001	0.5060	<.0001	0.6146	<.0001	0.7463
Histology	<.0001	0.9835	<.0001	0.3105	<.0001	0.0568	<.0001	0.7884
Comorbidity	<.0001	0.9968	<.0001	0.3816	<.0001	0.2220	<.0001	0.4960
Stage IV								
Previous Treatment			<.0001	0.7163	<.0001	0.9993	<.0001	1.0000
Sex	<.0001	0.9880	<.0001	0.9696	<.0001	0.3117	<.0001	0.6741
Age	<.0001	0.9999	<.0001	0.1806	<.0001	0.3759	<.0001	0.1899
SES(black)	<.0001	0.9968	0.0023	0.5961	0.0059	0.8120	0.0046	0.6906
SES(college)	<.0001	0.9998	<.0001	0.1793	<.0001	0.3275	0.0015	0.6617
SES(poverty)	<.0001	0.9963	<.0001	0.3311	<.0001	0.4743	<.0001	0.6781
Histology	<.0001	0.9834	<.0001	0.4588	<.0001	0.7163	<.0001	0.7388
Comorbidity	<.0001	0.9998	<.0001	0.9487	<.0001	0.5637	<.0001	0.4726
